# *cis*-Di­aqua­tetra­kis­(1-butyl-1*H*-imidazole-κ*N* ^3^)nickel(II) dichloride

**DOI:** 10.1107/S1600536813022496

**Published:** 2013-08-17

**Authors:** P. S. Kannan, A. S. Ganeshraja, K. Rajkumar, K. Anbalagan, A. SubbiahPandi

**Affiliations:** a Department of Physics, S. M. K. Fomra Institute of Technology, Thaiyur, Chennai 603 103, India; b Department of Chemistry, Pondicherry University, Pondicherry 605 014, India; c Department of Physics, Presidency College (Autonomous), Chennai 600 005, India

## Abstract

In the title compound, [Ni(C_7_H_12_N_2_)_4_(H_2_O)_2_]Cl_2_, the nickel(II) ion has a distorted octa­hedral coordination environment. It is surrounded by three N atoms and one O atom occupying the equatorial plane, and one N and one O atom in the axial positions. The imidazole ring systems are inclined to one another with dihedral angles varying between 38.3 (4) and 74.1 (4)°. In the crystal, mol­ecules are linked *via* O—H⋯Cl hydrogen bonds involving one Cl^−^ anion and the water mol­ecule in the equatorial plane, forming an inversion dimer-like arrangement. The water mol­ecule in the axial position is hydrogen-bonded to both Cl^−^ anions. There are also a number of C—H⋯Cl hydrogen bonds present, forming a three-dimensional structure. All four alkyl chains are disordered over two positions with refined occupancy ratios of 0.395 (15):0.605 (15), 0.658 (14):0.342 (14), 0.332 (11):0.668 (11) and 0.622 (12):0.378 (12).

## Related literature

For biological and pharmaceutical properties of imidazoles and imidazole-containing compounds, see: Roman *et al.* (2007 ▶); Nanterment *et al.* (2004 ▶); Congiu *et al.* (2008 ▶); Venkatesan *et al.* (2008 ▶); Bhatnagar *et al.* (2011 ▶); Puratchikody & Doble (2007 ▶); Gaonkar *et al.* (2009 ▶). For applications of imidazole and its derivatives in the construction of metal–organic frameworks, see: Huang *et al.* (2008 ▶, 2011 ▶).
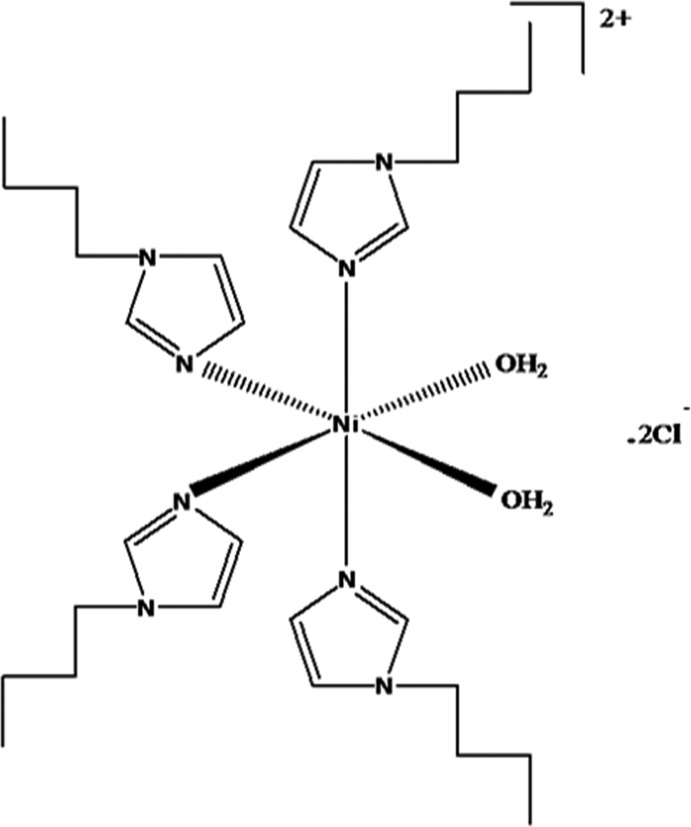


## Experimental

### 

#### Crystal data

[Ni(C_7_H_12_N_2_)_4_(H_2_O)_2_]Cl_2_
*M* *_r_* = 662.39
Monoclinic, 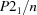

*a* = 8.533 (5) Å
*b* = 24.952 (5) Å
*c* = 17.641 (5) Å
β = 101.277 (5)°
*V* = 3684 (3) Å^3^
*Z* = 4
Mo *K*α radiation
μ = 0.71 mm^−1^
*T* = 293 K
0.30 × 0.30 × 0.25 mm

#### Data collection

Oxford Xcalibur diffractometer with Eos detector
Absorption correction: multi-scan (*CrysAlis PRO*; Oxford Diffraction, 2009 ▶) *T*_min_ = 0.790, *T*_max_ = 0.816
32409 measured reflections
6451 independent reflections
4027 reflections with *I* > 2σ(*I*)
*R*_int_ = 0.113

#### Refinement

*R*[*F* ^2^ > 2σ(*F* ^2^)] = 0.077
*wR*(*F* ^2^) = 0.233
*S* = 1.04
6451 reflections
530 parameters
707 restraints
H atoms treated by a mixture of independent and constrained refinement
Δρ_max_ = 0.77 e Å^−3^
Δρ_min_ = −0.69 e Å^−3^

### 

Data collection: *CrysAlis CCD* (Oxford Diffraction, 2009 ▶); cell refinement: *CrysAlis CCD*; data reduction: *CrysAlis RED* (Oxford Diffraction, 2009 ▶); program(s) used to solve structure: *SHELXS97* (Sheldrick, 2008 ▶); program(s) used to refine structure: *SHELXL97* (Sheldrick, 2008 ▶); molecular graphics: *ORTEP-3 for Windows* (Farrugia, 2012 ▶); software used to prepare material for publication: *SHELXL97* and *PLATON* (Spek, 2009 ▶).

## Supplementary Material

Crystal structure: contains datablock(s) global, I. DOI: 10.1107/S1600536813022496/su2624sup1.cif

Structure factors: contains datablock(s) I. DOI: 10.1107/S1600536813022496/su2624Isup2.hkl

Additional supplementary materials:  crystallographic information; 3D view; checkCIF report

## Figures and Tables

**Table 1:** Hydrogen-bond geometry (Å, °)

*D*—H⋯*A*	*D*—H	H⋯*A*	*D*⋯*A*	*D*—H⋯*A*
O1—H1*A*⋯Cl2^i^	0.84 (4)	2.35 (4)	3.165 (4)	164 (7)
O1—H1*B*⋯Cl1	0.84 (4)	2.37 (4)	3.185 (5)	166 (4)
O2—H2*A*⋯Cl2	0.86 (4)	2.43 (4)	3.250 (4)	160 (5)
O2—H2*B*⋯Cl2^i^	0.84 (6)	2.30 (5)	3.127 (4)	169 (5)
C3—H3⋯Cl2	0.93	2.67	3.593 (6)	171
C4—H4⋯Cl2	0.93	2.70	3.565 (8)	155
C5—H5⋯Cl1^ii^	0.93	2.72	3.640 (8)	173
C9—H9⋯Cl1	0.93	2.65	3.566 (7)	170
C10—H10⋯Cl1	0.93	2.67	3.562 (7)	160
C11—H11⋯Cl1^iii^	0.93	2.79	3.722 (7)	177

Symmetry codes: (i) 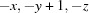; (ii) 
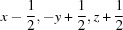
; (iii) 
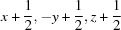
.

**Fig. 1.**
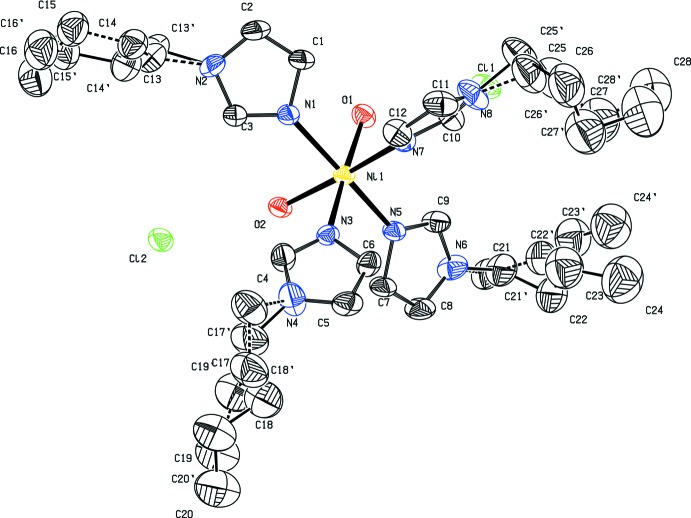
View of the molecular structure of the title compound, with atom labelling. The displacement ellipsoids are drawn at the 30% probability level. The H atoms have been omitted for clarity. The minor fraction of the disordered alkyl chains are indicated by dashed bonds and a suffix ' to the atom label.

**Fig. 2.**
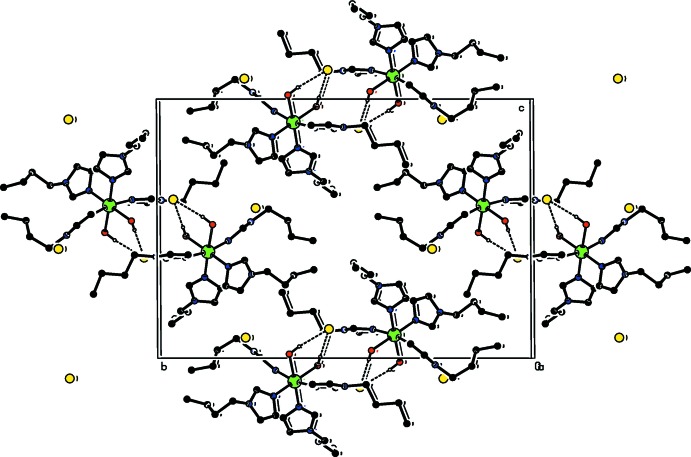
The crystal packing of the title compound viewed along the c axis. The O—H···Cl hydrogen bonds are shown as dashed lines [symmetry code (i): -x, -y+1, -z].

## supplementary crystallographic information

### 1. Comment

Imidazoles have been reported to serve as useful building blocks for the synthesis of diverse classes of bioactive molecules. In addition, imidazole-containing compounds exhibit a wide spectrum of pharmaceutical properties such as pesticides, fungicides, antibacterial, anti-inflammatory, anti-tubercular, anti-diabetic, antimalarial and antitumour (Roman *et al.*, 2007; Nanterment *et al.*, 2004; Congiu *et al.*, 2008; Venkatesan *et al.*, 2008; Bhatnagar *et al.*, 2011; Puratchikody & Doble 2007).

Knowledge of the detailed coordination behaviour of imidazoles and their limitation in the possible use in complexes with specific catalytic activity is of great current importance. Imidazoles, namely 1,3-diazacyclopenta-2,4-diene and its derivatives, have found a wide range of applications in coordination chemistry because of their multiple coordination modes as ligands to metal ions and for the construction of novel metal–organic frameworks (Huang *et al.*, 2008, 2011).

The chemistry of imidazole occupies an extremely important position within the family of five-membered heterocyclic compounds. Synthesis of imidazole derivatives has attracted great interest in recent years due to their broad spectrum of biological activities (Gaonkar *et al.*, 2009). This paper describes the synthesis and crystal structure of the nickel(II) complex of the imidazole ligand 1-butyl-1H-imidazole.

The molecular structure of the title compound is illustrated in Fig. 1. The nickel(II) ion has a distorted octahedral coordination environment. It is surrounded by four N and two O atoms; an N and an O atom are in the axial positions, and the other three N atoms and an O atom are in the equatorial plane. The imidazole ring (N1/N2/C1–C3) makes dihedral angles of 44.7 (4)°, 38.3 (4)° and 74.1 (4)° with the other three imidazole rings (N3/N4/C4–C6), (N5/N6/C7–C9) and (N7/N8/C10–C12), respectively.

In the crystal, molecules are linked via O—H···Cl hydrogen bonds, involving one Cl^-^ anion (Cl2) and the water molecule (O2) in the equatorial plane, to form an inversion dimer-like arrangement. The water molecule in the axial position (O1) is hydrogen-bonded to both Cl^-^ anions. There are a number of C—H···Cl interactions present forming a three-dimensional structure. Details are given in Table 1.

### 2. Experimental

NiCl_2_.6H_2_O (6.0 g) was dissolved in warm ethanol (3 ml). The solution was cooled in ice while adding slowly a solution of 1-butylimidazole (5.0 g in 5.6 ml EtOH); the reaction is quite exothermic. Cold ethanol (15 ml) was then added to initiate crystallization. The colourless crystalline solid was filtered and kept cold for 10 min then washed with two 5 ml portions of ethanol and dried in air.

### 3. Refinement

Thy water H atoms were located in a difference Fourier map and freely refined. The C-bound H atoms were included in calculated positions and treated as riding atoms: C—H = 0.93–0.97 Å with U_iso_(H) = 1.5U_eq_(C) for methyl and = 1.2U_eq_(C) for other H atoms. The four alkyl chains are disordered over two positions with refined occupancy ratios of 0.395 (15):0.605 (15); 0.658 (14):0.342 (14); 0.332 (11):0.668 (11); 0.622 (12):0.378 (12).

### Crystal data

**Table:**

[Ni(C_7_H_12_N_2_)_4_(H_2_O)_2_]Cl_2_	*F*(000) = 1416
*M**_r_* = 662.39	*D*_x_ = 1.194 Mg m^−^^3^
Monoclinic, *P*2_1_/*n*	Mo *K*α radiation, λ = 0.71073 Å
Hall symbol: -P 2yn	Cell parameters from 6451 reflections
*a* = 8.533 (5) Å	θ = 3.5–25.0°
*b* = 24.952 (5) Å	µ = 0.71 mm^−^^1^
*c* = 17.641 (5) Å	*T* = 293 K
β = 101.277 (5)°	Block, colourless
*V* = 3684 (3) Å^3^	0.30 × 0.30 × 0.25 mm
*Z* = 4	

### Data collection

**Table:**

Oxford Xcalibur diffractometer with Eos detector	6451 independent reflections
Radiation source: fine-focus sealed tube	4027 reflections with *I* > 2σ(*I*)
Graphite monochromator	*R*_int_ = 0.113
ω and φ scan	θ_max_ = 25.0°, θ_min_ = 3.5°
Absorption correction: multi-scan (*CrysAlis PRO*; Oxford Diffraction, 2009)	*h* = −10→10
*T*_min_ = 0.790, *T*_max_ = 0.816	*k* = −29→29
32409 measured reflections	*l* = −20→20

### Refinement

**Table:**

Refinement on *F*^2^	Primary atom site location: structure-invariant direct methods
Least-squares matrix: full	Secondary atom site location: difference Fourier map
*R*[*F*^2^ > 2σ(*F*^2^)] = 0.077	Hydrogen site location: inferred from neighbouring sites
*wR*(*F*^2^) = 0.233	H atoms treated by a mixture of independent and constrained refinement
*S* = 1.04	*w* = 1/[σ^2^(*F*_o_^2^) + (0.1168*P*)^2^ + 1.3167*P*] where *P* = (*F*_o_^2^ + 2*F*_c_^2^)/3
6451 reflections	(Δ/σ)_max_ = 0.001
530 parameters	Δρ_max_ = 0.77 e Å^−^^3^
707 restraints	Δρ_min_ = −0.69 e Å^−^^3^

### Special details

**Table:**

Geometry. Bond distances, angles etc. have been calculated using the rounded fractional coordinates. All su's are estimated from the variances of the (full) variance–covariance matrix. The cell esds are taken into account in the estimation of distances, angles and torsion angles
Refinement. Refinement of F^2^ against ALL reflections. The weighted R-factor wR and goodness of fit S are based on F^2^, conventional R-factors R are based on F, with F set to zero for negative F^2^. The threshold expression of F^2^ > 2sigma(F^2^) is used only for calculating R-factors(gt) etc. and is not relevant to the choice of reflections for refinement. R-factors based on F^2^ are statistically about twice as large as those based on F, and R-factors based on ALL data will be even larger.

### Fractional atomic coordinates and isotropic or equivalent isotropic displacement parameters (Å^2^)

**Table:**

	*x*	*y*	*z*	*U*_iso_*/*U*_eq_	Occ. (<1)
Ni1	0.11087 (7)	0.36734 (2)	0.08946 (4)	0.0436 (2)	
O1	0.2011 (5)	0.35389 (15)	−0.0154 (2)	0.0582 (12)	
O2	−0.0477 (4)	0.42750 (14)	0.0292 (2)	0.0524 (11)	
N1	0.2901 (5)	0.42634 (17)	0.1112 (3)	0.0510 (14)	
N2	0.4118 (6)	0.50391 (18)	0.1091 (3)	0.0670 (19)	
N3	0.0141 (5)	0.37746 (17)	0.1882 (3)	0.0485 (12)	
N4	−0.1056 (9)	0.4062 (2)	0.2792 (4)	0.103 (3)	
N5	−0.0736 (5)	0.31456 (17)	0.0473 (3)	0.0502 (14)	
N6	−0.2252 (6)	0.2556 (2)	−0.0259 (3)	0.0745 (17)	
N7	0.2726 (5)	0.31154 (17)	0.1466 (3)	0.0510 (14)	
N8	0.4466 (8)	0.2478 (3)	0.1766 (4)	0.093 (2)	
C1	0.4446 (6)	0.4179 (3)	0.1014 (4)	0.067 (2)	
C2	0.5172 (7)	0.4648 (3)	0.1000 (4)	0.075 (2)	
C3	0.2771 (6)	0.4780 (2)	0.1144 (4)	0.0590 (19)	
C4	−0.0356 (9)	0.4189 (3)	0.2200 (4)	0.075 (2)	
C5	−0.1008 (8)	0.3523 (3)	0.2850 (4)	0.077 (2)	
C6	−0.0246 (7)	0.3351 (2)	0.2305 (3)	0.064 (2)	
C7	−0.2319 (6)	0.3209 (2)	0.0543 (4)	0.067 (2)	
C8	−0.3219 (7)	0.2835 (3)	0.0117 (4)	0.077 (3)	
C9	−0.0765 (7)	0.2748 (3)	−0.0015 (4)	0.066 (2)	
C10	0.3326 (8)	0.2677 (2)	0.1224 (4)	0.069 (2)	
C11	0.4640 (8)	0.2782 (3)	0.2388 (4)	0.086 (3)	
C12	0.3546 (7)	0.3179 (3)	0.2213 (4)	0.071 (2)	
C13'	0.442 (3)	0.5613 (5)	0.1021 (11)	0.087 (3)	0.605 (15)
C14'	0.453 (2)	0.5875 (6)	0.1800 (10)	0.107 (3)	0.605 (15)
C15'	0.469 (3)	0.6486 (6)	0.1729 (10)	0.121 (4)	0.605 (15)
C16'	0.534 (3)	0.6707 (7)	0.2533 (10)	0.142 (5)	0.605 (15)
C17	−0.200 (2)	0.4441 (7)	0.3185 (11)	0.135 (4)	0.622 (12)
C18	−0.3681 (19)	0.4258 (7)	0.3220 (13)	0.147 (4)	0.622 (12)
C19	−0.439 (2)	0.4703 (8)	0.3679 (13)	0.168 (4)	0.622 (12)
C20	−0.6165 (19)	0.4634 (10)	0.3481 (14)	0.186 (6)	0.622 (12)
C21	−0.261 (3)	0.2083 (5)	−0.0753 (9)	0.101 (4)	0.658 (14)
C22	−0.284 (2)	0.1570 (5)	−0.0313 (10)	0.121 (4)	0.658 (14)
C23	−0.152 (3)	0.1463 (7)	0.0311 (13)	0.165 (4)	0.658 (14)
C24	−0.159 (4)	0.0888 (8)	0.0465 (15)	0.206 (7)	0.658 (14)
C25'	0.5629 (19)	0.2053 (6)	0.1663 (12)	0.143 (4)	0.668 (11)
C26'	0.530 (2)	0.1573 (7)	0.2172 (12)	0.173 (4)	0.668 (11)
C27'	0.370 (2)	0.1356 (7)	0.1799 (16)	0.195 (5)	0.668 (11)
C28'	0.425 (3)	0.0816 (7)	0.1589 (16)	0.224 (6)	0.668 (11)
C24'	−0.001 (5)	0.1041 (15)	0.054 (2)	0.186 (7)	0.342 (14)
C25	0.522 (4)	0.1950 (9)	0.177 (2)	0.143 (5)	0.332 (11)
C26	0.453 (4)	0.1712 (9)	0.1018 (19)	0.169 (5)	0.332 (11)
C27	0.345 (4)	0.1242 (14)	0.106 (3)	0.198 (5)	0.332 (11)
C28	0.407 (6)	0.0763 (11)	0.067 (3)	0.213 (7)	0.332 (11)
C19'	−0.374 (3)	0.4539 (16)	0.2983 (16)	0.161 (4)	0.378 (12)
C13	0.421 (5)	0.5635 (7)	0.1108 (17)	0.088 (4)	0.395 (15)
C14	0.518 (3)	0.5825 (8)	0.1868 (15)	0.093 (4)	0.395 (15)
C15	0.549 (3)	0.6419 (8)	0.1878 (18)	0.116 (4)	0.395 (15)
C16	0.419 (4)	0.6745 (9)	0.211 (2)	0.141 (6)	0.395 (15)
C17'	−0.086 (3)	0.4468 (10)	0.3464 (13)	0.127 (5)	0.378 (12)
C18'	−0.246 (3)	0.4415 (13)	0.3706 (14)	0.141 (4)	0.378 (12)
C22'	−0.208 (4)	0.1701 (10)	−0.0654 (16)	0.109 (4)	0.342 (14)
C20'	−0.532 (3)	0.4663 (18)	0.325 (2)	0.175 (6)	0.378 (12)
C21'	−0.289 (4)	0.2223 (9)	−0.0909 (15)	0.094 (4)	0.342 (14)
C23'	−0.094 (4)	0.1407 (13)	−0.006 (2)	0.145 (5)	0.342 (14)
Cl1	0.26436 (18)	0.23800 (7)	−0.07888 (10)	0.0737 (6)	
Cl2	−0.10336 (19)	0.54034 (6)	0.11212 (10)	0.0692 (6)	
H13D	0.54170	0.56650	0.08410	0.1040*	0.605 (15)
H14C	0.54450	0.57350	0.21600	0.1280*	0.605 (15)
H14D	0.35760	0.57930	0.20010	0.1280*	0.605 (15)
H15C	0.54210	0.65700	0.13860	0.1460*	0.605 (15)
H3	0.18350	0.49540	0.11980	0.0710*	
H4	−0.02410	0.45390	0.20370	0.0910*	
H5	−0.14240	0.33140	0.31990	0.0930*	
H6	−0.00100	0.29950	0.22230	0.0770*	
H7	−0.26970	0.34700	0.08390	0.0810*	
H8	−0.43060	0.27780	0.00870	0.0920*	
H9	0.01360	0.26140	−0.01740	0.0790*	
H10	0.29920	0.25280	0.07360	0.0830*	
H11	0.53630	0.27350	0.28510	0.1030*	
H12	0.33770	0.34530	0.25460	0.0850*	
H13C	0.35670	0.57740	0.06480	0.1040*	0.605 (15)
H20B	−0.64420	0.42820	0.36320	0.2790*	0.622 (12)
H20C	−0.65270	0.46770	0.29340	0.2790*	0.622 (12)
H21A	−0.17430	0.20280	−0.10290	0.1220*	0.658 (14)
H21B	−0.35730	0.21500	−0.11340	0.1220*	0.658 (14)
H22A	−0.38060	0.16010	−0.01070	0.1450*	0.658 (14)
H22B	−0.29650	0.12700	−0.06700	0.1450*	0.658 (14)
H23A	−0.16150	0.16690	0.07660	0.1980*	0.658 (14)
H23B	−0.05110	0.15550	0.01640	0.1980*	0.658 (14)
H24A	−0.07360	0.07920	0.08830	0.3090*	0.658 (14)
H24B	−0.14840	0.06910	0.00100	0.3090*	0.658 (14)
H24C	−0.25960	0.08050	0.06020	0.3090*	0.658 (14)
H25C	0.67160	0.21800	0.18280	0.1720*	0.668 (11)
H25D	0.54780	0.19460	0.11250	0.1720*	0.668 (11)
H26C	0.61160	0.13010	0.21940	0.2080*	0.668 (11)
H26D	0.52820	0.16930	0.26940	0.2080*	0.668 (11)
H27C	0.32160	0.15620	0.13480	0.2330*	0.668 (11)
H27D	0.29770	0.13330	0.21570	0.2330*	0.668 (11)
H28D	0.33350	0.06060	0.13580	0.3370*	0.668 (11)
H28E	0.48050	0.06390	0.20460	0.3370*	0.668 (11)
H28F	0.49460	0.08570	0.12280	0.3370*	0.668 (11)
H15D	0.36630	0.66450	0.15190	0.1460*	0.605 (15)
H16D	0.54950	0.70870	0.25020	0.2130*	0.605 (15)
H16E	0.63420	0.65380	0.27430	0.2130*	0.605 (15)
H16F	0.45910	0.66350	0.28620	0.2130*	0.605 (15)
H17A	−0.20670	0.47820	0.29180	0.1620*	0.622 (12)
H17B	−0.14210	0.44990	0.37080	0.1620*	0.622 (12)
H18A	−0.36570	0.39160	0.34820	0.1770*	0.622 (12)
H18B	−0.43180	0.42210	0.27030	0.1770*	0.622 (12)
H19A	−0.40010	0.46600	0.42300	0.2020*	0.622 (12)
H19B	−0.40850	0.50560	0.35270	0.2020*	0.622 (12)
H20A	−0.66650	0.48980	0.37500	0.2790*	0.622 (12)
H1	0.49060	0.38460	0.09660	0.0810*	
H1A	0.192 (9)	0.3804 (14)	−0.045 (3)	0.0870*	
H1B	0.208 (8)	0.3255 (12)	−0.040 (3)	0.0870*	
H2	0.62200	0.46990	0.09380	0.0910*	
H2A	−0.086 (7)	0.4568 (14)	0.043 (3)	0.0780*	
H2B	0.005 (7)	0.435 (2)	−0.005 (3)	0.0780*	
H13A	0.31420	0.57830	0.10350	0.1060*	0.395 (15)
H13B	0.47010	0.57590	0.06880	0.1060*	0.395 (15)
H14A	0.46130	0.57360	0.22780	0.1120*	0.395 (15)
H14B	0.61920	0.56370	0.19700	0.1120*	0.395 (15)
H15A	0.64810	0.64920	0.22340	0.1400*	0.395 (15)
H15B	0.56150	0.65300	0.13670	0.1400*	0.395 (15)
H16A	0.44560	0.71180	0.21030	0.2120*	0.395 (15)
H16B	0.40690	0.66440	0.26200	0.2120*	0.395 (15)
H16C	0.32010	0.66810	0.17520	0.2120*	0.395 (15)
H17C	0.00100	0.43710	0.38810	0.1530*	0.378 (12)
H17D	−0.06950	0.48290	0.32920	0.1530*	0.378 (12)
H18C	−0.25400	0.46670	0.41170	0.1690*	0.378 (12)
H18D	−0.26020	0.40550	0.38890	0.1690*	0.378 (12)
H19C	−0.38840	0.42330	0.26360	0.1930*	0.378 (12)
H19D	−0.34210	0.48440	0.27090	0.1930*	0.378 (12)
H20D	−0.61750	0.46860	0.28090	0.2630*	0.378 (12)
H20E	−0.52210	0.49980	0.35240	0.2630*	0.378 (12)
H20F	−0.55430	0.43830	0.35870	0.2630*	0.378 (12)
H21C	−0.25940	0.23540	−0.13800	0.1130*	0.342 (14)
H21D	−0.40410	0.21930	−0.09850	0.1130*	0.342 (14)
H22C	−0.30070	0.14710	−0.06940	0.1310*	0.342 (14)
H22D	−0.16480	0.16150	−0.11090	0.1310*	0.342 (14)
H23C	−0.07040	0.17210	0.02640	0.1740*	0.342 (14)
H23D	−0.01100	0.14130	−0.03670	0.1740*	0.342 (14)
H24D	0.09440	0.12200	0.07930	0.2790*	0.342 (14)
H24E	0.02790	0.07220	0.02890	0.2790*	0.342 (14)
H24F	−0.06480	0.09460	0.09060	0.2790*	0.342 (14)
H25A	0.63720	0.19850	0.18240	0.1720*	0.332 (11)
H25B	0.49950	0.17310	0.21870	0.1720*	0.332 (11)
H26A	0.53850	0.15980	0.07680	0.2030*	0.332 (11)
H26B	0.39220	0.19850	0.06950	0.2030*	0.332 (11)
H27A	0.23660	0.13270	0.08050	0.2380*	0.332 (11)
H27B	0.34370	0.11590	0.16000	0.2380*	0.332 (11)
H28A	0.34400	0.04520	0.07330	0.3180*	0.332 (11)
H28B	0.51640	0.06980	0.09100	0.3180*	0.332 (11)
H28C	0.39930	0.08380	0.01340	0.3180*	0.332 (11)

### Atomic displacement parameters (Å^2^)

**Table:**

	*U*^11^	*U*^22^	*U*^33^	*U*^12^	*U*^13^	*U*^23^
Ni1	0.0345 (4)	0.0485 (4)	0.0474 (4)	0.0012 (3)	0.0071 (3)	0.0018 (3)
O1	0.063 (2)	0.060 (2)	0.055 (2)	0.010 (2)	0.020 (2)	0.0009 (18)
O2	0.0412 (19)	0.059 (2)	0.058 (2)	0.0088 (17)	0.0125 (17)	0.0086 (18)
N1	0.038 (2)	0.052 (2)	0.062 (3)	−0.0019 (18)	0.007 (2)	−0.007 (2)
N2	0.051 (3)	0.064 (3)	0.085 (4)	−0.018 (2)	0.011 (3)	−0.001 (3)
N3	0.045 (2)	0.052 (2)	0.050 (2)	−0.0002 (19)	0.013 (2)	0.0101 (19)
N4	0.161 (6)	0.079 (4)	0.092 (4)	0.027 (4)	0.080 (4)	0.021 (3)
N5	0.034 (2)	0.056 (2)	0.059 (3)	0.0009 (18)	0.005 (2)	−0.004 (2)
N6	0.061 (3)	0.094 (3)	0.066 (3)	−0.027 (3)	0.006 (3)	−0.025 (3)
N7	0.043 (2)	0.058 (3)	0.048 (2)	0.0035 (19)	−0.001 (2)	0.006 (2)
N8	0.086 (4)	0.105 (4)	0.089 (4)	0.052 (4)	0.018 (4)	0.027 (3)
C1	0.039 (3)	0.071 (3)	0.095 (5)	−0.002 (3)	0.020 (3)	−0.009 (3)
C2	0.043 (3)	0.088 (4)	0.099 (5)	−0.014 (3)	0.023 (4)	−0.004 (4)
C3	0.045 (3)	0.058 (3)	0.074 (4)	−0.001 (2)	0.012 (3)	−0.002 (3)
C4	0.106 (5)	0.061 (3)	0.070 (4)	0.006 (4)	0.044 (4)	0.009 (3)
C5	0.076 (4)	0.098 (4)	0.066 (4)	0.005 (4)	0.033 (4)	0.032 (3)
C6	0.072 (4)	0.059 (3)	0.062 (4)	−0.001 (3)	0.017 (3)	0.019 (3)
C7	0.033 (3)	0.076 (4)	0.092 (5)	0.000 (3)	0.009 (3)	−0.011 (3)
C8	0.036 (3)	0.098 (5)	0.094 (5)	−0.010 (3)	0.003 (3)	−0.012 (4)
C9	0.050 (3)	0.086 (4)	0.064 (4)	−0.010 (3)	0.018 (3)	−0.022 (3)
C10	0.075 (4)	0.074 (4)	0.059 (3)	0.018 (3)	0.013 (3)	−0.001 (3)
C11	0.050 (4)	0.127 (6)	0.073 (4)	0.013 (4)	−0.006 (3)	0.020 (4)
C12	0.065 (4)	0.086 (4)	0.055 (3)	0.007 (3)	−0.002 (3)	−0.001 (3)
C13'	0.084 (7)	0.081 (5)	0.098 (6)	−0.017 (5)	0.021 (6)	0.007 (5)
C14'	0.116 (7)	0.094 (5)	0.109 (6)	−0.015 (6)	0.015 (6)	−0.010 (5)
C15'	0.129 (8)	0.097 (6)	0.135 (7)	−0.003 (7)	0.018 (7)	−0.022 (6)
C16'	0.147 (10)	0.115 (8)	0.154 (9)	−0.012 (8)	0.007 (9)	−0.032 (7)
C17	0.149 (7)	0.150 (7)	0.124 (7)	0.016 (6)	0.075 (6)	−0.004 (6)
C18	0.138 (7)	0.154 (8)	0.150 (8)	0.006 (6)	0.028 (6)	−0.007 (6)
C19	0.151 (7)	0.193 (8)	0.169 (8)	0.019 (7)	0.052 (7)	−0.008 (7)
C20	0.153 (9)	0.219 (12)	0.193 (12)	0.015 (10)	0.053 (10)	0.011 (11)
C21	0.099 (7)	0.100 (6)	0.100 (6)	−0.025 (6)	0.008 (6)	−0.036 (5)
C22	0.130 (7)	0.100 (5)	0.133 (7)	−0.020 (6)	0.024 (6)	−0.035 (5)
C23	0.170 (8)	0.150 (7)	0.171 (8)	0.010 (7)	0.025 (6)	0.006 (7)
C24	0.215 (13)	0.174 (9)	0.220 (12)	0.001 (10)	0.019 (11)	0.039 (10)
C25'	0.140 (7)	0.142 (7)	0.153 (8)	0.068 (5)	0.044 (6)	0.022 (6)
C26'	0.184 (8)	0.138 (6)	0.194 (8)	0.045 (6)	0.030 (7)	0.029 (6)
C27'	0.194 (8)	0.174 (8)	0.213 (9)	0.027 (6)	0.033 (7)	0.001 (7)
C28'	0.241 (11)	0.187 (9)	0.234 (12)	0.035 (9)	0.021 (10)	−0.018 (9)
C24'	0.187 (12)	0.170 (11)	0.190 (11)	0.010 (10)	0.012 (10)	0.021 (10)
C25	0.147 (9)	0.128 (7)	0.156 (9)	0.056 (6)	0.033 (7)	0.021 (6)
C26	0.169 (9)	0.162 (8)	0.177 (9)	0.033 (7)	0.034 (8)	−0.008 (7)
C27	0.201 (9)	0.184 (9)	0.206 (10)	0.011 (7)	0.032 (8)	0.000 (7)
C28	0.226 (14)	0.183 (10)	0.220 (14)	0.012 (11)	0.023 (12)	−0.013 (11)
C19'	0.154 (7)	0.173 (8)	0.158 (8)	0.009 (7)	0.037 (6)	−0.006 (7)
C13	0.091 (8)	0.081 (5)	0.094 (8)	−0.019 (5)	0.020 (7)	0.007 (6)
C14	0.089 (8)	0.091 (6)	0.098 (7)	−0.011 (6)	0.016 (6)	−0.008 (6)
C15	0.119 (8)	0.100 (6)	0.126 (8)	−0.013 (6)	0.014 (7)	−0.012 (6)
C16	0.151 (11)	0.119 (9)	0.150 (11)	0.012 (9)	0.021 (10)	−0.014 (9)
C17'	0.140 (8)	0.138 (8)	0.112 (8)	0.019 (8)	0.047 (7)	−0.016 (7)
C18'	0.144 (7)	0.156 (8)	0.134 (8)	0.016 (7)	0.053 (6)	−0.028 (7)
C22'	0.117 (8)	0.102 (7)	0.108 (8)	−0.011 (7)	0.023 (7)	−0.032 (6)
C20'	0.151 (9)	0.203 (11)	0.176 (12)	0.014 (11)	0.043 (9)	−0.002 (11)
C21'	0.091 (8)	0.096 (7)	0.092 (8)	−0.025 (6)	0.008 (6)	−0.026 (6)
C23'	0.152 (9)	0.134 (8)	0.145 (9)	0.001 (7)	0.020 (7)	−0.007 (7)
Cl1	0.0533 (9)	0.0964 (12)	0.0703 (10)	0.0051 (8)	0.0091 (8)	−0.0347 (9)
Cl2	0.0697 (10)	0.0651 (9)	0.0807 (11)	0.0139 (7)	0.0345 (9)	0.0187 (8)

### Geometric parameters (Å, º)

**Table:**

Ni1—O1	2.164 (4)	C12—H12	0.9300
Ni1—O2	2.157 (4)	C13—H13B	0.9700
Ni1—N1	2.103 (5)	C13—H13A	0.9700
Ni1—N3	2.084 (5)	C13'—H13D	0.9700
Ni1—N5	2.075 (5)	C13'—H13C	0.9700
Ni1—N7	2.077 (5)	C14—H14A	0.9700
O1—H1B	0.84 (4)	C14—H14B	0.9700
O1—H1A	0.84 (4)	C14'—H14D	0.9700
O2—H2A	0.86 (4)	C14'—H14C	0.9700
O2—H2B	0.84 (6)	C15—H15B	0.9700
N1—C1	1.379 (7)	C15—H15A	0.9700
N1—C3	1.296 (7)	C15'—H15C	0.9700
N2—C3	1.338 (7)	C15'—H15D	0.9700
N2—C13'	1.465 (14)	C16—H16B	0.9600
N2—C13	1.489 (18)	C16—H16C	0.9600
N2—C2	1.358 (8)	C16—H16A	0.9600
N3—C4	1.287 (9)	C16'—H16D	0.9600
N3—C6	1.371 (7)	C16'—H16E	0.9600
N4—C17	1.497 (19)	C16'—H16F	0.9600
N4—C4	1.339 (10)	C17—H17A	0.9700
N4—C17'	1.54 (2)	C17—H17B	0.9700
N4—C5	1.349 (9)	C17'—H17C	0.9700
N5—C7	1.389 (7)	C17'—H17D	0.9700
N5—C9	1.311 (9)	C18—H18A	0.9700
N6—C8	1.349 (8)	C18—H18B	0.9700
N6—C9	1.346 (8)	C18'—H18C	0.9700
N6—C21	1.463 (15)	C18'—H18D	0.9700
N6—C21'	1.43 (3)	C19—H19A	0.9700
N7—C12	1.375 (9)	C19—H19B	0.9700
N7—C10	1.314 (7)	C19'—H19C	0.9700
N8—C10	1.320 (10)	C19'—H19D	0.9700
N8—C25	1.47 (3)	C20—H20C	0.9600
N8—C11	1.318 (10)	C20—H20A	0.9600
N8—C25'	1.488 (18)	C20—H20B	0.9600
C1—C2	1.327 (10)	C20'—H20D	0.9600
C5—C6	1.333 (9)	C20'—H20F	0.9600
C7—C8	1.342 (9)	C20'—H20E	0.9600
C11—C12	1.355 (10)	C21—H21B	0.9700
C13—C14	1.51 (4)	C21—H21A	0.9700
C13'—C14'	1.51 (2)	C21'—H21C	0.9700
C14—C15	1.51 (3)	C21'—H21D	0.9700
C14'—C15'	1.54 (2)	C22—H22A	0.9700
C15—C16	1.50 (4)	C22—H22B	0.9700
C15'—C16'	1.52 (2)	C22'—H22C	0.9700
C17—C18	1.52 (2)	C22'—H22D	0.9700
C17'—C18'	1.51 (4)	C23—H23A	0.9700
C18—C19	1.56 (3)	C23—H23B	0.9700
C18'—C19'	1.54 (4)	C23'—H23C	0.9700
C19—C20	1.50 (3)	C23'—H23D	0.9700
C19'—C20'	1.54 (4)	C24—H24A	0.9600
C21—C22	1.53 (2)	C24—H24B	0.9600
C21'—C22'	1.50 (4)	C24—H24C	0.9600
C22—C23	1.44 (3)	C24'—H24D	0.9600
C22'—C23'	1.48 (4)	C24'—H24E	0.9700
C23—C24	1.46 (3)	C24'—H24F	0.9500
C23'—C24'	1.50 (5)	C25—H25A	0.9700
C25—C26	1.47 (5)	C25—H25B	0.9700
C25'—C26'	1.56 (3)	C25'—H25D	0.9700
C26—C27	1.50 (4)	C25'—H25C	0.9700
C26'—C27'	1.50 (3)	C26—H26B	0.9700
C27—C28	1.53 (6)	C26—H26A	0.9700
C27'—C28'	1.50 (3)	C26'—H26C	0.9700
C1—H1	0.9300	C26'—H26D	0.9700
C2—H2	0.9300	C27—H27B	0.9800
C3—H3	0.9300	C27—H27A	0.9700
C4—H4	0.9300	C27'—H27D	0.9700
C5—H5	0.9300	C27'—H27C	0.9700
C6—H6	0.9300	C28—H28A	0.9600
C7—H7	0.9300	C28—H28C	0.9500
C8—H8	0.9300	C28—H28B	0.9600
C9—H9	0.9300	C28'—H28E	0.9600
C10—H10	0.9300	C28'—H28F	0.9600
C11—H11	0.9300	C28'—H28D	0.9600
			
O1—Ni1—O2	88.88 (14)	C16'—C15'—H15D	110.00
O1—Ni1—N1	84.08 (17)	H15C—C15'—H15D	108.00
O1—Ni1—N3	176.94 (16)	C15—C16—H16B	110.00
O1—Ni1—N5	88.11 (17)	C15—C16—H16C	109.00
O1—Ni1—N7	89.84 (17)	H16A—C16—H16B	110.00
O2—Ni1—N1	88.08 (16)	H16A—C16—H16C	109.00
O2—Ni1—N3	90.84 (16)	H16B—C16—H16C	109.00
O2—Ni1—N5	84.62 (16)	C15—C16—H16A	110.00
O2—Ni1—N7	177.31 (16)	H16E—C16'—H16F	109.00
N1—Ni1—N3	98.95 (18)	C15'—C16'—H16D	110.00
N1—Ni1—N5	169.4 (2)	C15'—C16'—H16E	110.00
N1—Ni1—N7	89.44 (17)	C15'—C16'—H16F	109.00
N3—Ni1—N5	88.84 (19)	H16D—C16'—H16E	109.00
N3—Ni1—N7	90.56 (19)	H16D—C16'—H16F	110.00
N5—Ni1—N7	97.70 (17)	H17A—C17—H17B	108.00
H1A—O1—H1B	111 (5)	N4—C17—H17A	108.00
Ni1—O1—H1A	114 (4)	N4—C17—H17B	108.00
Ni1—O1—H1B	130 (4)	C18—C17—H17A	109.00
Ni1—O2—H2A	134 (4)	C18—C17—H17B	108.00
Ni1—O2—H2B	98 (4)	C18'—C17'—H17C	111.00
H2A—O2—H2B	108 (5)	C18'—C17'—H17D	111.00
Ni1—N1—C1	123.3 (4)	N4—C17'—H17D	112.00
Ni1—N1—C3	129.7 (4)	N4—C17'—H17C	112.00
C1—N1—C3	104.4 (5)	H17C—C17'—H17D	109.00
C2—N2—C13'	124.1 (11)	C17—C18—H18A	111.00
C2—N2—C3	105.0 (5)	C17—C18—H18B	111.00
C3—N2—C13	121.6 (17)	H18A—C18—H18B	109.00
C2—N2—C13	133.4 (17)	C19—C18—H18B	111.00
C3—N2—C13'	130.3 (11)	C19—C18—H18A	111.00
C4—N3—C6	104.3 (5)	C19'—C18'—H18C	110.00
Ni1—N3—C6	122.6 (3)	C17'—C18'—H18D	111.00
Ni1—N3—C4	132.8 (4)	C17'—C18'—H18C	111.00
C4—N4—C17'	116.5 (11)	C19'—C18'—H18D	110.00
C5—N4—C17	127.3 (9)	H18C—C18'—H18D	109.00
C4—N4—C17	125.1 (8)	H19A—C19—H19B	109.00
C4—N4—C5	106.5 (6)	C18—C19—H19A	110.00
C5—N4—C17'	126.8 (10)	C18—C19—H19B	110.00
Ni1—N5—C9	129.2 (4)	C20—C19—H19A	111.00
Ni1—N5—C7	125.3 (4)	C20—C19—H19B	111.00
C7—N5—C9	104.5 (5)	C18'—C19'—H19C	110.00
C8—N6—C21'	121.3 (14)	C20'—C19'—H19D	110.00
C8—N6—C9	106.8 (5)	C18'—C19'—H19D	110.00
C8—N6—C21	129.2 (11)	C20'—C19'—H19C	110.00
C9—N6—C21'	130.4 (15)	H19C—C19'—H19D	108.00
C9—N6—C21	123.5 (11)	C19—C20—H20A	109.00
Ni1—N7—C10	131.6 (5)	H20B—C20—H20C	109.00
C10—N7—C12	104.5 (5)	C19—C20—H20B	109.00
Ni1—N7—C12	123.6 (4)	C19—C20—H20C	110.00
C10—N8—C25	127.0 (15)	H20A—C20—H20B	109.00
C10—N8—C25'	126.5 (10)	H20A—C20—H20C	109.00
C10—N8—C11	109.5 (7)	C19'—C20'—H20F	109.00
C11—N8—C25'	122.9 (10)	H20D—C20'—H20E	110.00
C11—N8—C25	122.4 (15)	C19'—C20'—H20E	109.00
N1—C1—C2	109.2 (6)	C19'—C20'—H20D	110.00
N2—C2—C1	108.0 (5)	H20E—C20'—H20F	109.00
N1—C3—N2	113.3 (5)	H20D—C20'—H20F	110.00
N3—C4—N4	112.7 (6)	N6—C21—H21B	109.00
N4—C5—C6	106.2 (6)	N6—C21—H21A	109.00
N3—C6—C5	110.4 (5)	H21A—C21—H21B	108.00
N5—C7—C8	109.3 (5)	C22—C21—H21A	109.00
N6—C8—C7	107.2 (5)	C22—C21—H21B	109.00
N5—C9—N6	112.0 (5)	N6—C21'—H21D	112.00
N7—C10—N8	110.9 (6)	H21C—C21'—H21D	110.00
N8—C11—C12	105.7 (6)	C22'—C21'—H21C	112.00
N7—C12—C11	109.4 (6)	C22'—C21'—H21D	112.00
N2—C13—C14	110.5 (19)	N6—C21'—H21C	112.00
N2—C13'—C14'	109.1 (13)	C21—C22—H22A	109.00
C13—C14—C15	113 (2)	C21—C22—H22B	109.00
C13'—C14'—C15'	110.3 (13)	C23—C22—H22A	109.00
C14—C15—C16	114 (2)	C23—C22—H22B	109.00
C14'—C15'—C16'	107.8 (14)	H22A—C22—H22B	108.00
N4—C17—C18	115.3 (14)	C21'—C22'—H22C	100.00
N4—C17'—C18'	101.0 (18)	C21'—C22'—H22D	100.00
C17—C18—C19	105.8 (14)	C23'—C22'—H22C	100.00
C17'—C18'—C19'	106 (2)	C23'—C22'—H22D	100.00
C18—C19—C20	105.9 (17)	H22C—C22'—H22D	104.00
C18'—C19'—C20'	108 (2)	H23A—C23—H23B	108.00
N6—C21—C22	113.9 (12)	C22—C23—H23A	111.00
N6—C21'—C22'	101 (2)	C22—C23—H23B	111.00
C21—C22—C23	112.6 (15)	C24—C23—H23A	111.00
C21'—C22'—C23'	148 (3)	C24—C23—H23B	111.00
C22—C23—C24	105.7 (19)	C24'—C23'—H23C	93.00
C22'—C23'—C24'	170 (3)	C24'—C23'—H23D	93.00
N8—C25—C26	105 (2)	C22'—C23'—H23D	93.00
N8—C25'—C26'	106.2 (13)	C22'—C23'—H23C	93.00
C25—C26—C27	114 (3)	H23C—C23'—H23D	103.00
C25'—C26'—C27'	106.1 (16)	C23—C24—H24A	110.00
C26—C27—C28	109 (3)	C23—C24—H24B	109.00
C26'—C27'—C28'	97.5 (16)	H24A—C24—H24C	110.00
N1—C1—H1	125.00	H24B—C24—H24C	110.00
C2—C1—H1	125.00	C23—C24—H24C	109.00
N2—C2—H2	126.00	H24A—C24—H24B	109.00
C1—C2—H2	126.00	C23'—C24'—H24F	110.00
N1—C3—H3	123.00	H24E—C24'—H24F	110.00
N2—C3—H3	123.00	H24D—C24'—H24E	109.00
N3—C4—H4	124.00	H24D—C24'—H24F	110.00
N4—C4—H4	124.00	C23'—C24'—H24D	109.00
N4—C5—H5	127.00	C23'—C24'—H24E	109.00
C6—C5—H5	127.00	N8—C25—H25B	111.00
N3—C6—H6	125.00	C26—C25—H25A	110.00
C5—C6—H6	125.00	C26—C25—H25B	111.00
N5—C7—H7	125.00	H25A—C25—H25B	109.00
C8—C7—H7	125.00	N8—C25—H25A	111.00
N6—C8—H8	126.00	H25C—C25'—H25D	109.00
C7—C8—H8	126.00	N8—C25'—H25D	111.00
N5—C9—H9	124.00	N8—C25'—H25C	111.00
N6—C9—H9	124.00	C26'—C25'—H25C	110.00
N7—C10—H10	124.00	C26'—C25'—H25D	110.00
N8—C10—H10	125.00	C25—C26—H26B	109.00
N8—C11—H11	127.00	C25—C26—H26A	109.00
C12—C11—H11	127.00	H26A—C26—H26B	108.00
N7—C12—H12	125.00	C27—C26—H26A	109.00
C11—C12—H12	125.00	C27—C26—H26B	108.00
N2—C13—H13B	109.00	C25'—C26'—H26C	111.00
C14—C13—H13A	110.00	C27'—C26'—H26D	110.00
C14—C13—H13B	110.00	H26C—C26'—H26D	109.00
H13A—C13—H13B	108.00	C25'—C26'—H26D	110.00
N2—C13—H13A	109.00	C27'—C26'—H26C	111.00
H13C—C13'—H13D	108.00	H27A—C27—H27B	108.00
N2—C13'—H13D	110.00	C28—C27—H27A	110.00
N2—C13'—H13C	110.00	C28—C27—H27B	110.00
C14'—C13'—H13C	110.00	C26—C27—H27A	110.00
C14'—C13'—H13D	110.00	C26—C27—H27B	110.00
C13—C14—H14B	109.00	C26'—C27'—H27C	112.00
C13—C14—H14A	109.00	C26'—C27'—H27D	112.00
H14A—C14—H14B	108.00	C28'—C27'—H27C	112.00
C15—C14—H14A	109.00	C28'—C27'—H27D	112.00
C15—C14—H14B	109.00	H27C—C27'—H27D	110.00
C13'—C14'—H14C	110.00	C27—C28—H28B	109.00
C15'—C14'—H14D	109.00	C27—C28—H28C	109.00
H14C—C14'—H14D	108.00	H28A—C28—H28B	109.00
C13'—C14'—H14D	110.00	H28A—C28—H28C	110.00
C15'—C14'—H14C	110.00	H28B—C28—H28C	110.00
H15A—C15—H15B	108.00	C27—C28—H28A	109.00
C16—C15—H15A	109.00	H28E—C28'—H28F	110.00
C16—C15—H15B	109.00	C27'—C28'—H28D	109.00
C14—C15—H15A	109.00	C27'—C28'—H28E	109.00
C14—C15—H15B	109.00	C27'—C28'—H28F	109.00
C14'—C15'—H15C	110.00	H28D—C28'—H28E	109.00
C14'—C15'—H15D	110.00	H28D—C28'—H28F	110.00
C16'—C15'—H15C	110.00		
			
O1—Ni1—N1—C1	−45.5 (5)	C6—N3—C4—N4	−0.9 (8)
O2—Ni1—N1—C1	−134.6 (5)	C4—N3—C6—C5	1.9 (7)
N3—Ni1—N1—C1	134.9 (5)	Ni1—N3—C4—N4	173.1 (5)
N7—Ni1—N1—C1	44.4 (5)	Ni1—N3—C6—C5	−172.8 (4)
O1—Ni1—N1—C3	113.5 (6)	C5—N4—C4—N3	−0.5 (9)
O2—Ni1—N1—C3	24.4 (6)	C17—N4—C4—N3	−168.7 (10)
N3—Ni1—N1—C3	−66.1 (6)	C4—N4—C5—C6	1.6 (8)
N7—Ni1—N1—C3	−156.6 (6)	C4—N4—C17—C18	125.2 (14)
O2—Ni1—N3—C4	−37.4 (6)	C5—N4—C17—C18	−41 (2)
N1—Ni1—N3—C4	50.8 (6)	C17—N4—C5—C6	169.5 (11)
N5—Ni1—N3—C4	−122.0 (6)	C7—N5—C9—N6	0.1 (7)
N7—Ni1—N3—C4	140.3 (6)	Ni1—N5—C9—N6	−168.8 (4)
O2—Ni1—N3—C6	135.6 (4)	Ni1—N5—C7—C8	171.7 (4)
N1—Ni1—N3—C6	−136.2 (4)	C9—N5—C7—C8	2.2 (7)
N5—Ni1—N3—C6	51.0 (4)	C21—N6—C8—C7	175.2 (9)
N7—Ni1—N3—C6	−46.7 (4)	C9—N6—C21—C22	99.0 (18)
O1—Ni1—N5—C7	−140.3 (5)	C21—N6—C9—N5	−174.5 (8)
O2—Ni1—N5—C7	−51.3 (5)	C8—N6—C9—N5	−2.3 (8)
N3—Ni1—N5—C7	39.7 (5)	C8—N6—C21—C22	−71.4 (19)
N7—Ni1—N5—C7	130.1 (5)	C9—N6—C8—C7	3.6 (8)
O1—Ni1—N5—C9	26.5 (6)	C12—N7—C10—N8	−1.1 (7)
O2—Ni1—N5—C9	115.5 (6)	Ni1—N7—C10—N8	172.4 (5)
N3—Ni1—N5—C9	−153.5 (6)	Ni1—N7—C12—C11	−172.8 (4)
N7—Ni1—N5—C9	−63.1 (6)	C10—N7—C12—C11	1.4 (7)
O1—Ni1—N7—C10	−32.4 (5)	C10—N8—C11—C12	0.5 (8)
N1—Ni1—N7—C10	−116.4 (5)	C11—N8—C25'—C26'	76.8 (14)
N3—Ni1—N7—C10	144.6 (5)	C25'—N8—C11—C12	168.7 (10)
N5—Ni1—N7—C10	55.7 (6)	C25'—N8—C10—N7	−167.3 (9)
O1—Ni1—N7—C12	140.1 (5)	C11—N8—C10—N7	0.4 (9)
N1—Ni1—N7—C12	56.0 (5)	C10—N8—C25'—C26'	−117.1 (14)
N3—Ni1—N7—C12	−42.9 (5)	N1—C1—C2—N2	0.2 (8)
N5—Ni1—N7—C12	−131.8 (5)	N4—C5—C6—N3	−2.2 (8)
Ni1—N1—C1—C2	164.1 (5)	N5—C7—C8—N6	−3.7 (8)
C3—N1—C1—C2	0.6 (8)	N8—C11—C12—N7	−1.2 (8)
Ni1—N1—C3—N2	−163.3 (4)	N2—C13'—C14'—C15'	−174.7 (16)
C1—N1—C3—N2	−1.3 (8)	C13'—C14'—C15'—C16'	−161.5 (19)
C13'—N2—C2—C1	−172.9 (10)	N4—C17—C18—C19	177.8 (14)
C13'—N2—C3—N1	172.7 (11)	C17—C18—C19—C20	160.4 (17)
C3—N2—C2—C1	−1.0 (7)	N6—C21—C22—C23	−53 (2)
C2—N2—C3—N1	1.5 (8)	C21—C22—C23—C24	−158.1 (19)
C2—N2—C13'—C14'	−113.4 (15)	N8—C25'—C26'—C27'	68.5 (17)
C3—N2—C13'—C14'	76.8 (19)	C25'—C26'—C27'—C28'	115.7 (18)

### Hydrogen-bond geometry (Å, º)

**Table:**

*D*—H···*A*	*D*—H	H···*A*	*D*···*A*	*D*—H···*A*
O1—H1*A*···Cl2^i^	0.84 (4)	2.35 (4)	3.165 (4)	164 (7)
O1—H1*B*···Cl1	0.84 (4)	2.37 (4)	3.185 (5)	166 (4)
O2—H2*A*···Cl2	0.86 (4)	2.43 (4)	3.250 (4)	160 (5)
O2—H2*B*···Cl2^i^	0.84 (6)	2.30 (5)	3.127 (4)	169 (5)
C3—H3···Cl2	0.93	2.67	3.593 (6)	171
C4—H4···Cl2	0.93	2.70	3.565 (8)	155
C5—H5···Cl1^ii^	0.93	2.72	3.640 (8)	173
C9—H9···Cl1	0.93	2.65	3.566 (7)	170
C10—H10···Cl1	0.93	2.67	3.562 (7)	160
C11—H11···Cl1^iii^	0.93	2.79	3.722 (7)	177

Symmetry codes: (i) −*x*, −*y*+1, −*z*; (ii) *x*−1/2, −*y*+1/2, *z*+1/2; (iii) *x*+1/2, −*y*+1/2, *z*+1/2.
